# Enhancing the Performance of Laser Powder Bed-Fused Inconel 718 Through Effective Spatter Removal via Atmosphere Protection System Optimization

**DOI:** 10.3390/ma19050917

**Published:** 2026-02-27

**Authors:** Yuxuan Jiang, Yin Wang, Yukai Chen, Yu Lu, Chuyue Wen, Bin Han, Qi Zhang

**Affiliations:** School of Mechanical Engineering, Xi’an Jiaotong University, Xi’an 710049, China; jiang20000801@stu.xjtu.edu.cn (Y.J.); kingyin@stu.xjtu.edu.cn (Y.W.); a364694998@stu.xjtu.edu.cn (Y.C.); luyu9910@stu.xjtu.edu.cn (Y.L.); 15605293306@163.com (C.W.); hanbinghost@mail.xjtu.edu.cn (B.H.)

**Keywords:** laser powder bed fusion, spatter particle removal, atmosphere protection system, the mechanical properties of laser powder bed fused Inconel 718

## Abstract

While extensive research on laser powder bed fusion has focused on optimizing process parameters to improve the performance of manufactured parts, the critical role of effective spatter particle removal in mitigating defects during manufacturing has not received commensurate attention. To address these issues, this study investigates the influence of a key parameter in the atmosphere protection system, namely, airflow velocity, on part performance. Methodologically, a combined approach of numerical simulation and experimental methods was employed to examine in detail the effect of airflow velocity on spatter removal efficiency and its corresponding contribution to the enhancement of formed Inconel 718 part quality. First, Computational Fluid Dynamics–Discrete Phase Model simulations identified an optimal airflow velocity of 0.57 m/s. Subsequently, experimental observations using a high-speed camera system revealed that velocities below 0.6 m/s led to spatter redeposition, resulting in pore and defect formation, whereas velocities exceeding 0.6 m/s increased spatter size and reduced molten-pool stability. The simulation and experimental results are consistent, demonstrating that an appropriate airflow velocity can effectively suppress defects and thereby improve the quality of the fabricated components. This research provides a viable pathway for significantly enhancing the mechanical properties of laser powder bed-fused Inconel 718.

## 1. Introduction

Additive manufacturing (AM) technology has experienced rapid development in the past decade, owing to its unparalleled design freedom and the ability to rapidly manufacture complex prototypes. Among the various metal additive manufacturing techniques, laser powder bed fusion (L-PBF) is widely employed [1,2]. Owing to its high customization and flexibility, L-PBF holds significant potential for application in industries such as aerospace and automotive [3,4,5,6].

Fundamentally, L-PBF employs a high-energy laser beam as the energy source to melt metal powder. However, the high-energy laser beam induces the generation of spatter dust. The spatter generated during L-PBF can take various forms, and different process parameters significantly influence its behavior [7]. The formation of spatter is primarily attributed to the instability in the molten pool, and its size is significantly larger than that of the powder used [8]. And the spatter can absorb the incident laser energy along the laser path, potentially leading to laser attenuation. Additionally, the spatter condensed from the surface of the molten metal can be sprayed and re-deposited onto the surface of the printed part. These re-deposited particles can significantly increase the layer thickness [9]. If the powder layer thickness exceeds the critical value, defects such as porosity and insufficient fusion may occur. Therefore, these spatters can have a significant impact on the quality of the printed part [10]. The importance of spatter removal is further amplified in additive subtractive hybrid manufacturing (ASHM) processes, where L-PBF is combined with subtractive manufacturing techniques such as milling and drilling [11]. In ASHM, the residual spatter on the printed parts can lead to increased surface roughness [12], accelerated tool wear, and dimensional inaccuracies during subtractive operations. Effective spatter removal is thus critical to ensure the precision and quality of hybrid manufactured components [13]. Therefore, it is essential to thoroughly investigate these factors to identify optimal printing conditions and implement measures to mitigate these negative effects.

Currently, the primary method for spatter removal involves the protective airflow generated by the atmosphere protection system [14]. It has been reported that spatter removal can enhance the microstructure and mechanical properties of the printed part [15]. The airflow velocity significantly influences the spatter removal efficiency, so the entrainment removal effect of airflow and airflow velocity on spatter should be carefully considered during L-PBF [16]. Concurrently, during the printing process, it is essential to ensure the stable operation of the atmosphere protection system to provide adequate protective airflow and prevent fluctuations that may compromise print quality.

Methodologically, given the high experimental costs, studying the effect of airflow on spatter through simulation is a feasible approach. Philo et al. [17] developed a coupled Computational Fluid Dynamics (CFD)–Discrete Phase Model (DPM) numerical simulation framework to investigate the interaction between airflow and spatters in L-PBF. Their findings revealed that structural parameters of the shielding gas inlet and outlet in the forming cavity, such as the inlet nozzle radius and the inlet/outlet heights, significantly influenced airflow velocity, uniformity, and spatter concentration. Chien et al. [18] further optimized the computational CFD-DPM simulation framework to investigate the interaction between the airflow field and ejected spatter during a single scan. Tang et al. [19] developed the MYSINT 3147D printer and optimized the dust removal system design to achieve a stable and uniform airflow field, thereby enhancing spatter dust removal efficiency. Sun et al. [20] conducted a simulation of the airflow field within the L-PBF forming cavity, achieving the first optimization of the airflow field structure. They employed the particle tracer method to assess the airflow field in their experiment. The results demonstrated that the structural optimization enhanced the uniformity of the airflow field distribution and improved the airflow’s ability to entrain spatter. Zhang et al. [21] introduced a novel design for the dust removal system within the forming cavity, aimed at enhancing spatter removal efficiency. Additionally, numerous scholars have conducted extensive numerical simulations and experimental studies on basic process parameters. Their analyses focused on the effects of various basic process parameters, including laser power, scanning speed, laser diameter, powder layer thickness, laser beam mode, scanning strategy, and forming cavity pressure, on spatter generation [22,23,24,25,26].

In L-PBF processes, the airflow velocity of the atmosphere protection system is also a critical parameter that governs spatter removal efficiency. However, limited research has been conducted on the impact of airflow velocity on spatter removal. This results from several contributing factors. Firstly, airflow velocity is strongly coupled with oxygen content. In most existing studies, it is treated merely as an adjustment means to achieve low oxygen levels, rather than as an independent process variable. Secondly, at the industrial and equipment manufacturer level, the role of protective gas is often simplified into a binary performance metric: as long as the final oxygen content meets the required standard, the atmosphere system is considered functional. Equipment manuals typically specify only the use of high-purity inert gas and the maintenance of a low-oxygen environment, rarely providing guidance on an optimal flow velocity range. Currently, studies on airflow velocity primarily focus on the influence of airflow uniformity on the performance of L-PBF-formed parts. Ladewig et al. [27] investigated the primary process by-products of L-PBF, including spatter, and examined how inert airflow uniformity affects the quality consistency of parts in large-scale L-PBF processes, such as mechanical properties. They concluded that the uniformity of airflow and part positioning in the powder bed significantly impacts part quality consistency, which is crucial for large-scale L-PBF applications. Shen et al. [28] analyzed samples produced under low airflow velocity conditions and found increased porosity and fusion defects, suggesting that spatter significantly affects powder bed stability, uniformity, and laser attenuation.

Collectively, the influence of shielding airflow velocity in laser powder bed fusion equipment on the overall printing process has not yet been fully and clearly elucidated. Existing studies often focus either solely on the impact of airflow velocity on spatter removal efficiency or only on the properties of the formed parts, without systematically investigating the spatter behavior during the printing process itself. To address this research gap, this study employs the widely used Inconel 718 alloy, critical for high-temperature components, as the target material. Combining numerical simulations with experimental methods, we systematically investigate the effects of airflow velocity on the motion and removal of spatter particles and their consequent impact on the quality of L-PBF-fabricated Inconel 718 parts. Following a rigorous and comprehensive research pathway—from simulation and optimization to experimental investigation and performance enhancement—this work provides a novel strategy for further improving the performance of components manufactured via laser powder bed fusion.

## 2. Simulation and Experimental Methods

### 2.1. CFD-DPM Theory

The transport of spatter particles by inert gas was simulated to study the spatter removal function of the atmosphere protection system, which was considered as a gas-solid flow. Considering that the motion of particles is usually controlled by lift and resistance forces, this system is classified as a dilute flow [29]. In contrast, dense flow systems typically experience particle collisions, which influence particle motion. The Euler–Lagrange algorithm was employed, combining the Eulerian and Lagrangian approaches. Within the Euler–Lagrange framework, CFD is employed to model the gas flow, accounting for the conservation of mass and momentum. Simultaneously, spatter particles are represented using the Discrete Phase Model (DPM), which considers the conservation of momentum equations.

In CFD simulations, considering the airflow field within the L-PBF chamber, it is unnecessary to include the powder bed or laser source in the calculations. Additionally, to minimize computational costs while maintaining an acceptable level of accuracy, the working fluid (argon) within the chamber was assumed to behave as an ideal incompressible Newtonian fluid with constant material properties [30]. Furthermore, the effects of heat transfer were neglected, and the heat source simulation was excluded. The fluid domain was treated as a continuum, and DPM was employed to track particles through the flow field. Coupling was utilized to describe the interaction between particles and fluids [31]. For fluid flows with bounded walls and small average pressure gradients, the turbulent *k*-*ε* model was applied. The governing equation for turbulent kinetic energy, which enables the *k*-*ε* model, is as follows [32]:

(1) $$\frac{\partial }{\partial t}\left(\rho k\right)+\frac{\partial }{\partial x_{j}}\left(\rho k\mu_{j}\right)=\frac{\partial }{\partial x_{j}}\left[\left(\mu +\frac{\mu_{t}}{\sigma_{k}}\right)\frac{\partial k}{\partial x_{j}}\right]+G_{k}+G_{b}-\rho \varepsilon$$

Energy dissipation transfer equation:

(2) $$\frac{\partial }{\partial t}\left(\rho \varepsilon \right)+\frac{\partial }{\partial x_{j}}\left(\rho \varepsilon \mu_{j}\right)=\frac{\partial }{\partial x_{j}}\left[\left(\mu +\frac{\mu_{t}}{\sigma_{\varepsilon }}\right)\frac{\partial \varepsilon }{\partial x_{j}}\right]+\rho C_{1}S\varepsilon -\rho C_{2}\frac{\varepsilon^{2}}{k+\sqrt{v\varepsilon }}-C_{1\varepsilon }\frac{\varepsilon }{k}C_{3\varepsilon }G_{b}$$

However, buoyancy can be ignored in this airflow simulation:

(3) $$C_{1}=\max\left[0.43,\frac{\eta }{\eta +5}\right]$$

(4) $$\eta =S\frac{k}{\varepsilon }$$

(5) $$S=\sqrt{2S_{ij}S_{ij}}$$

where ρ is the density of the working fluid, t is the time, k is the turbulence kinetic energy, ε is the turbulence dissipation rate, $G_{k}$ is the turbulent kinetic energy (*k*) generated by the mean velocity gradient, $G_{b}$ is the turbulent kinetic energy (*k*) generated by buoyancy, $\mu_{t}$ is the eddy viscosity, $C_{1\varepsilon }$ is the constant with the value 1.44, $C_{2}$ is the constant with the value 1.9, $C_{3\varepsilon }$ is the degree to which ε is affected by the buoyancy of the fluid flow, S is modulus of the average strain rate tensor, $\sigma_{k}$ is the turbulent Prandtl number, and $\sigma_{\varepsilon }$ is also the turbulent Prandtl number.

The CFD simulation parameters used in this paper are shown in Table 1 below, mainly including the basic parameters of oxygen, argon and the cavity. At the same time, Table 1 also shows the meanings of other parameters in the above formula. Notably, an isothermal assumption was implemented in the model with the objective of achieving a tractable computational cost. These parameter values were specified within ANSYS Fluent 2020R1.

In this study, DPM model was used to track particles in the whole flow field by Lagrange method. DPM model is suitable for simulating the flow of dilute particles [33,34]. In the Cartesian coordinate system, the force acting on particles is considered in the force balance equation, and the following formula is given [35]:

(6) $$\frac{du_{p}}{dt}=\frac{u-u_{p}}{\tau_{p}}+\frac{g\left(\rho_{p}-\rho \right)}{\rho_{p}}+F$$

where $u_{p}$ is the particle velocity, $\rho_{p}$ is the particle density, u is the molecular viscosity of the fluid, $\tau_{p}$ is the particle relaxation time, and *F* is the external force on the particle.

The first item on the right of the equal sign is the resistance per unit particle mass, the second item is gravity, and the last item is additional force, such as virtual mass, rotation, electrostatic or magnetic force.

The particle relaxation time is defined as follows:

(7) $$\tau_{P}=\frac{\rho_{p}d_{p}^{2}}{18\mu }\frac{24}{C_{D}Re}$$

For smooth particles, the drag coefficient $C_{D}$ is as follows:

(8) $$C_{D}=a_{1}+\frac{a_{2}}{Re}+\frac{a_{3}}{Re}$$

*Re* is the relative Reynolds number, and the main calculation formula is as follows:

(9) $$Re=\frac{\rho d_{p}\left|u_{P}-u\right|}{\mu }$$

The DPM simulation parameters are detailed in Table 2. These parameters include the density, diameter, generation rate, primary generation location, and initial speed of spatter particles. Additionally, Table 2 provides the definitions of other parameters used in the governing equations. All parameter values were set in Fluent.

### 2.2. Model Construction

**Figure 1:**
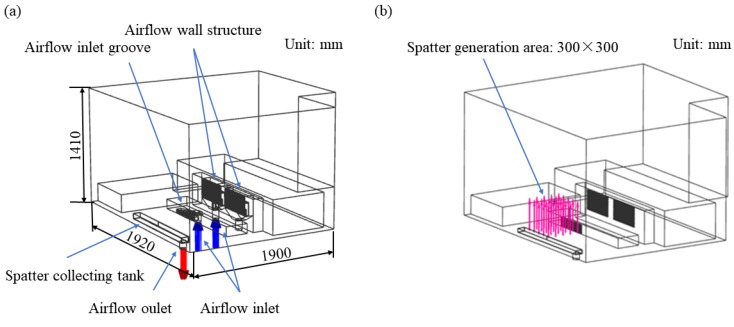
Simulation model: (**a**) size and composition, (**b**) the initial position of the spatter.

Based on the self-developed L-PBF equipment ZJ-300 (Xi’an Jiaotong University, Xi’an, Shaanxi, China), the simulation model for this study was established. Generally, it is observed that certain components, such as the laser galvanometer, have minimal impact on spatter removal [36]. Consequently, the simulation model incorporated the cavity, airflow inlet groove, airflow wall structure, and spatter collection tank. The cavity dimensions were 1900 mm in length, 1920 mm in width, and 1410 mm in height. After the simplification described above, the simulation model, as depicted in Figure 1a, was developed for the spatter removal system under investigation.

During airflow velocity simulations, the influence of spatter particles was incorporated by integrating spatter particle parameters into the fluid simulation. In the L-PBF process, spattering typically occurs above the substrate. Consequently, a square area measuring 300 mm on each side was designated as the spatter generation zone, consistent with the actual substrate size.

Simulation parameters, including spatter density, particle diameter, and generation rate, are presented in Table 2. The initial spatter generation location is illustrated in Figure 1b. And high-purity argon was used as the flowing gas within the cavity, with simulation parameters set as specified in Table 1 and Table 2. At the same time, in the actual L-PBF equipment, only one airflow wall structure operates. Consequently, to simplify the model, only the left airflow wall structure was retained in this simulation. In the simulation, the boundary conditions were defined as follows: a pressure inlet at 3.5 MPa with a turbulence intensity of 5%, a pressure outlet, and adiabatic smooth walls. Finally, mesh independence was verified by monitoring the outlet pressure and the internal airflow velocity. Both parameters stabilized when the mesh count exceeded 1.5 million.

### 2.3. Airflow Uniformity Test

**Figure 2:**
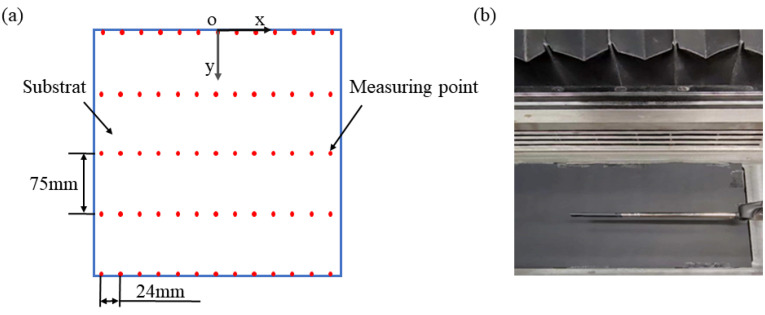
Schematic diagram of measuring airflow velocity: (**a**) measuring points for airflow velocity, (**b**) airflow velocity measurement using the hot-wire anemometer.

To analyze the airflow uniformity across the substrate surface, 65 points (5 × 13) were strategically selected for airflow velocity measurements. Figure 2a illustrates the schematic layout of airflow velocity measuring points for both simulation and experimental setups. Figure 2b depicts the schematic representation of airflow velocity measurements conducted on the substrate surface using the hot-wire anemometer (Fluke Electronics and Instrument Company, Everett, Washington, USA). And the hot-wire anemometer was calibrated under the experimental conditions.

### 2.4. High-Speed Microscopic Imaging System

**Figure 3:**
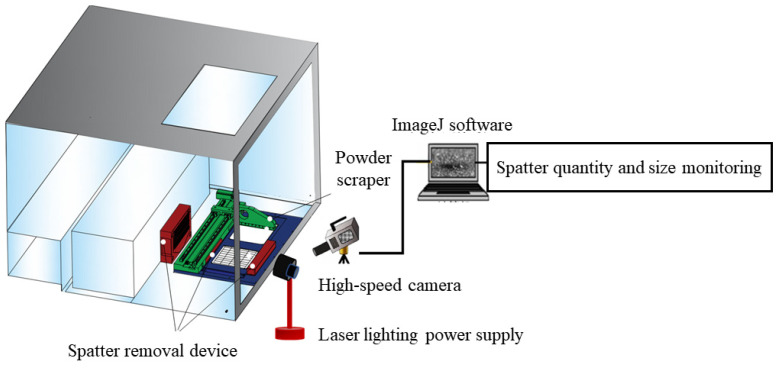
Schematic diagram of the built high-speed microscopic imaging system.

In order to observe the effect of airflow velocity on spatter removal during the experiment, a high-speed microscopic imaging system was developed, as shown in Figure 3. The system primarily consisted of a high-speed camera, a laser lighting power supply, and ImageJ software (version 1.54g). The high-speed camera (HF Agile Device Co., Ltd., Hefei, Anhui, China) featured a full-frame resolution of 1920 × 1080, with a maximum acquisition speed of 16,900 fps. Equipped with a global shutter and automatic exposure functionality, the system’s minimum exposure time was 100 ns, enabling single-channel L-PBF process capture. Furthermore, to enhance the observation of spatter during the L-PBF process, the high-speed microscopic imaging system’s lens was positioned at a 30° angle relative to the substrate. The laser lighting power supply utilized 5500 K standard white light, primarily functioning to illuminate the forming chamber and enhance the imaging quality of the high-speed microscopic system. Spatter particles were identified and analyzed for their quantity and size using ImageJ image analysis software (version 1.54g).

### 2.5. Sample Processing and Related Experiments

#### 2.5.1. Material

In this study, Inconel 718 powder with particle diameters ranging from 11 to 83 μm was selected for sample printing. The detailed chemical composition percentages of the powder are provided by the manufacturer (Tianjin Liz Technology Co., Ltd., Tianjin, China) and presented in Table 3.

#### 2.5.2. Microstructure Analysis

The sample was initially set using the XQ-2B setting machine (Shine Instrument Co., Ltd., Shanghai, China) and subsequently polished with the MPD-1A automatic polishing machine (Shaoxing Penghui Technology Co., Ltd., Shaoxing, Zhejiang, China) until a smooth and uniform surface was achieved. Finally, a Nikon metallographic microscope (Nikon Precision Co., Ltd., Shanghai, China) was employed to examine defects, such as pores.

#### 2.5.3. Densification Analysis

The electronic densification tester selected for the test is model QL-300D (Midea Electronics Technology Co., Ltd., Xiamen, Fujian, China), with a measuring range of 0–300 g, weighing accuracy of 0.005 g, and density accuracy of 0.001 g/cm^3^. Prior to measurement, the sample surface was polished to remove oxidation and contaminants, followed by ultrasonic cleaning and drying. To minimize experimental error, three samples were selected per group, each measured five times, with the average value recorded.

#### 2.5.4. Hardness Analysis

In this study, the hardness of Inconel 718 blocks (dimensions: 8 × 8 × 8 mm) fabricated via the L-PBF process was measured using the micro-Vickers hardness tester HVT-1000A (Shanghai Hongce Instrument Technology Co., Ltd., Shanghai, China). To minimize experimental error, three blocks were selected per experimental parameter group. Five distinct areas were measured for each block, and the average Vickers hardness value was calculated. The hardness testing equipment has a measuring range of 5–3000 HV, with loading test forces of 0.09807, 0.2452, 0.4904, 0.9807, 1.961, 2.942, 4.904, and 9.807 N. The minimum detection unit is 0.025 μm, and the maximum allowable sample height is 75 mm.

#### 2.5.5. Tensile Property Analysis

**Figure 4:**
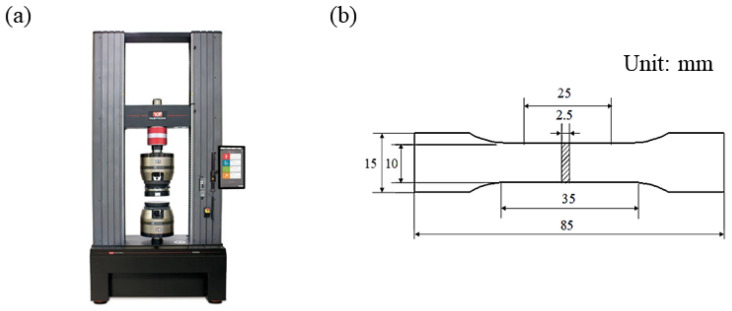
Tensile performance test diagram: (**a**) universal material testing machine Instron 5982, (**b**) sample size diagram for tensile property test.

The universal material testing machine Instron 5982 (Instron Test Equipment Trading Co., Ltd., Shanghai, China), as shown in Figure 4a, was used to carry out tensile property tests, and the size of standard tensile parts is shown in Figure 4b. For each group of experimental parameters, three samples were printed. Tensile strength, yield strength, and elongation were chosen as evaluation criteria. The average value of the three samples was computed as the final evaluation metric.

### 2.6. Evaluation Index of Airflow Velocity

The study of airflow velocity primarily aims to ensure comprehensive removal of soot spatter, thereby guaranteeing the surface quality and mechanical properties of the final formed part [37,38]. Consequently, the main evaluation criteria for airflow velocity in this study encompass: airflow uniformity above the substrate under the influence of the dust removal system, spatter removal efficiency, surface quality, internal microstructural pores, and the mechanical performance of the formed part.

## 3. Results and Discussion

Through numerical simulations analyzing the effect of airflow velocity on spatter, the optimal airflow velocity has been determined. Based on the optimal process parameters of L-PBF, the effects of varying airflow velocities on spatter, sample microstructure, and mechanical properties were investigated.

### 3.1. Simulation Results

According to the design function of the airflow wall and intake structure, this study selected different groups of airflow velocities for the airflow wall ($v_{a}$) and inlet structure ($v_{i}$). Due to the functional differences between the airflow wall and inlet structure, the airflow wall velocity parameter was designed to be higher than that of the inlet structure. The average airflow velocity at the substrate surface ($v_{s}$), as presented in Table 4, was selected because the L-PBF process initiates from this plane, making it a representative measure for the build surface. This velocity was obtained as a spatial average from CFD simulations conducted in Fluent, with the specific measurement points corresponding to the locations illustrated in Figure 2. The following airflow velocity refers to the average airflow velocity of the substrate surface.

**Figure 5:**
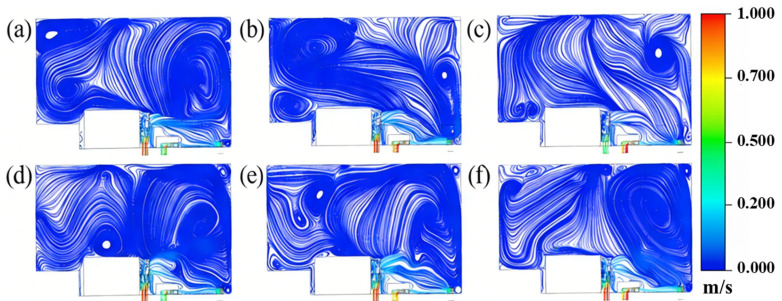
Airflow streamline diagrams of different schemes: (**a**) 0.26 m/s, (**b**) 0.46 m/s, (**c**) 0.78 m/s, (**d**) 0.57 m/s, (**e**) 0.74 m/s, (**f**) 0.92 m/s.

Select the middle interface of the substrate to get the airflow streamline diagrams of different schemes, as shown in Figure 5.

**Figure 6:**
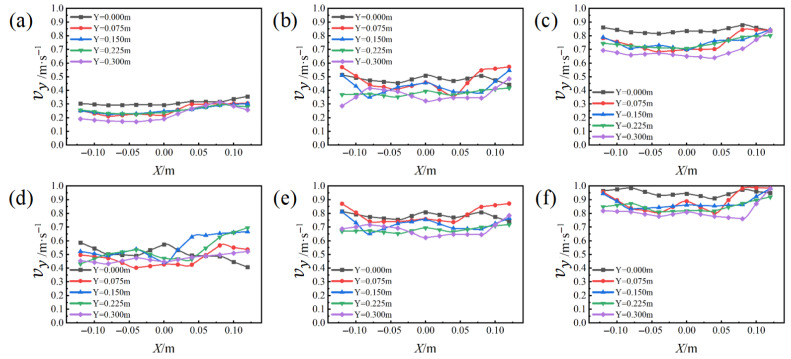
Simulation results of airflow velocity uniformity of different schemes: (**a**) 0.26 m/s, (**b**) 0.46 m/s, (**c**) 0.78 m/s, (**d**) 0.57 m/s, (**e**) 0.74 m/s, (**f**) 0.92 m/s.

The effects of airflow at different velocities can be observed through streamline diagrams. Concurrently, the simulation and analysis of airflow uniformity within the cavity were conducted, yielding the airflow velocity above the substrate surface ($v_{y}$), as depicted in Figure 6. The measurement points selected for airflow velocity are illustrated in Figure 2.

Based on the simulation results, the standard deviation of airflow velocity for the six airflow scheme groups was calculated, as presented in Table 5. The analysis indicates that airflow velocity has a minimal impact on the uniformity of the forming cavity, with the structural design of the atmosphere protection system being the primary influencing factor. The standard deviation of airflow velocity across the six schemes is notably low, all below the cavity’s designed airflow uniformity standard deviation of 0.1, indicating a relatively uniform airflow distribution.

The optimization of airflow velocity aims to enhance the removal of soot spatter, minimize its generation and deposition, thereby ensuring the quality of the formed parts. Consequently, in the airflow velocity optimization simulation, it is essential to incorporate the flow field simulation of spatter effects to comprehensively assess the advantages and disadvantages of different airflow velocities. This approach facilitates better control over spatter issues during processing and improves the quality of the formed parts.

**Figure 7:**
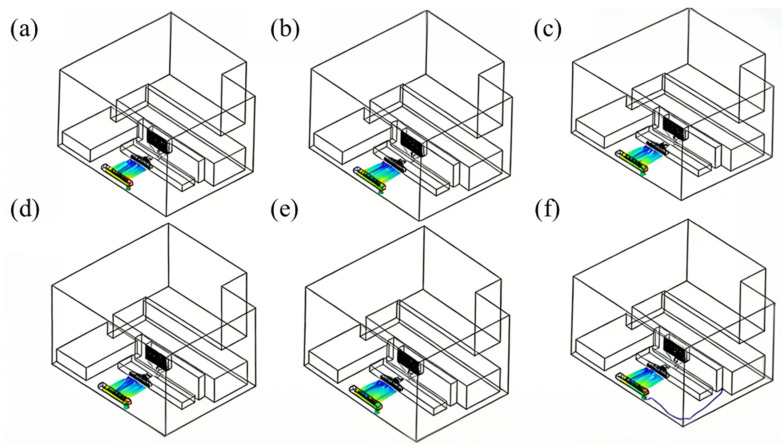
Trajectory of spatter at different airflow velocities: (**a**) 0.26 m/s, (**b**) 0.46 m/s, (**c**) 0.78 m/s, (**d**) 0.57 m/s, (**e**) 0.74 m/s, (**f**) 0.92 m/s.

Spatter particles were integrated into the flow field simulation, and the trajectories of spatter under six distinct airflow velocity schemes are observed, as illustrated in Figure 7. And in this figure, the different colors represent initial particles from different locations. Simulation results reveal that the improper selection of airflow velocity can lead to spatter being discharged from the side or upper sections of the exhaust structure, resulting in disordered movement within the cavity. This disrupts the efficiency of spatter dust removal and negatively impacts the quality of L-PBF-formed parts.

**Figure 8:**
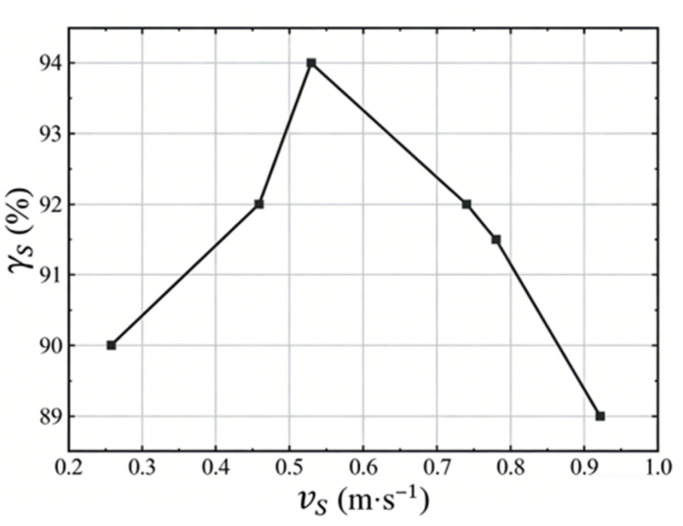
Removal rate of spatter at different airflow velocities.

The spatter removal rate ($r_{s}$) is significantly influenced by the average airflow velocity at the substrate surface, as demonstrated in Figure 8. This figure illustrates the relationship between the average airflow velocity and the spatter removal rate. Initially, the removal rate increases proportionally with the airflow velocity. However, beyond a certain threshold, higher velocities can disrupt the powder bed, destabilize the molten pool, and consequently decrease the removal efficiency. The optimal spatter removal rate is achieved at an airflow velocity of 0.57 m/s.

Based on the simulation results, it is evident that maintaining an airflow velocity within the range of 0.46~0.78 m/s ensures both the uniformity of the airflow across the substrate surface and an optimal spatter removal effect. Consequently, these findings guided subsequent experimental work to validate and further refine the airflow velocity parameters for enhanced process quality.

### 3.2. Experimental Results

#### 3.2.1. The Effect of Airflow Velocity on Spatter

The airflow velocity significantly influences both the generation and removal of spatter in the L-PBF process. Spatter redeposition can lead to insufficient molten pool melting and the formation of pores, thereby affecting the quality of the final formed parts. Building upon the airflow simulation results, this study conducted single-channel L-PBF spatter observation experiments under varying airflow velocities.

The experimental airflow velocities were set at 0, 0.15, 0.3, 0.45, 0.6, and 0.75 m/s, as detailed in Table 6. And the airflow velocity of 0 m/s serves as the control group for this study. At the same time, the high-speed microscopic observation system was utilized to perform the single-channel spatter observation experiments. The experiments employed optimized L-PBF process parameters, including a laser power of 210 W, scanning speed of 900 mm/s, and scanning spacing of 0.09 mm, with Inconel 718 powder as the material.

**Figure 9:**
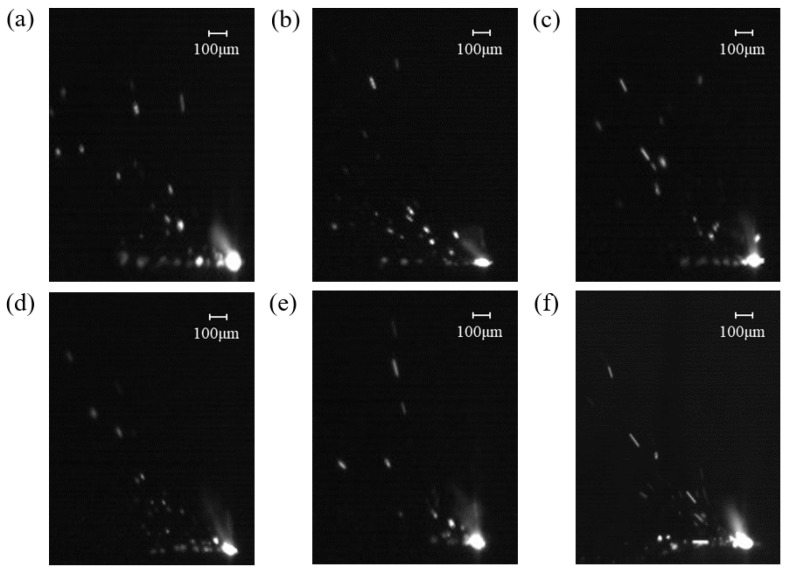
Spatter images of different airflow velocities detected by high-speed camera: (**a**) 0 m/s, (**b**) 0.15 m/s, (**c**) 0.3 m/s, (**d**) 0.45 m/s, (**e**) 0.6 m/s, (**f**) 0.75 m/s.

To mitigate the influence of powder layer thickness and single-pass forming position on spatter generation, the initial powder bed thickness was slightly thicker than 0.2 mm. After each single-pass L-PBF forming process, the molten powder was prevented from adhering to the substrate. Subsequently, the substrate was raised by 0.1 mm, and the powder spreading scraper removed the single molten pool formed by the melting and solidification of powder under the high-energy laser’s influence. Finally, the base plate was lowered by 0.1 mm, and the powder scraper was reapplied to ensure consistent powder layer thickness. Single-channel L-PBF experiments were conducted at the same location but with varying airflow velocities. Figure 9 illustrates the spatter captured by the high-speed camera system under different airflow velocities. In this experimental setup, the airflow velocity referred to the average airflow velocity at the substrate surface.

**Figure 10:**
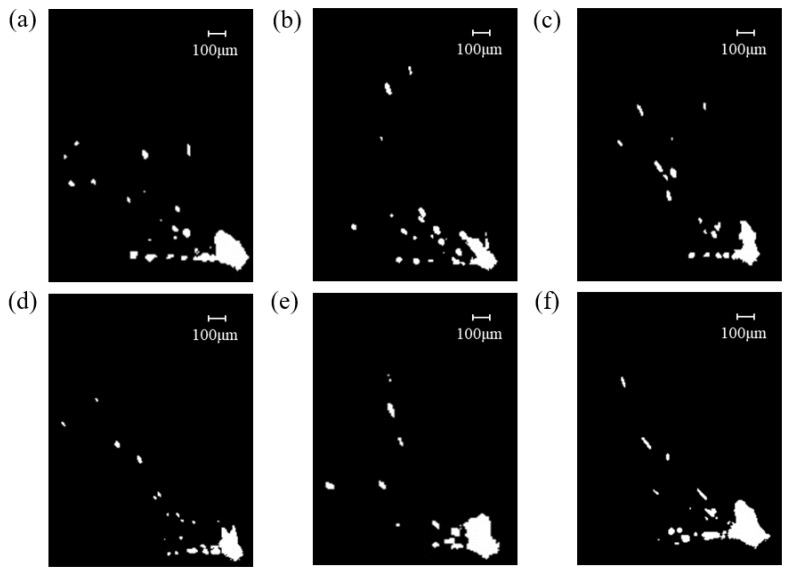
Spatter images at different airflow velocities after binarization: (**a**) 0 m/s, (**b**) 0.15 m/s, (**c**) 0.3 m/s, (**d**) 0.45 m/s, (**e**) 0.6 m/s, (**f**) 0.75 m/s.

Then, perform binarization to obtain clearer spatter images, as shown in Figure 10.

**Figure 11:**
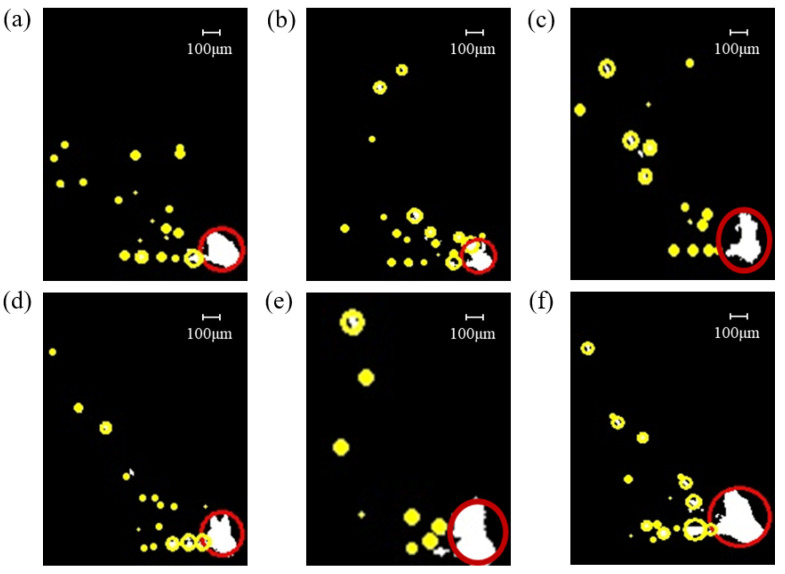
Spatter images at different airflow velocities after ImageJ processing: (**a**) 0 m/s, (**b**) 0.15 m/s, (**c**) 0.3 m/s, (**d**) 0.45 m/s, (**e**) 0.6 m/s, (**f**) 0.75 m/s.

ImageJ software was employed to process binary images, identifying spatter particles under different airflow velocities, as illustrated in Figure 11. Metal vapor appears as diffuse, irregular, large-area bright zones in cloud-like or banded shapes, typically concentrated near the molten pool or along specific airflow paths. Spatter particles appear as discrete, relatively well-defined, isolated bright spots with approximately circular or elliptical shapes, which are smaller in size and randomly distributed across the field of view. Therefore, circularity is used as the core criterion for differentiation: spatter particles are more spherical with higher circularity, while metal vapor regions are irregular with lower circularity. In the images, yellow circles denoted spatter particles, while red circles indicated metal vapor generated by the interaction of the laser with metal powder and the molten pool. Based on this analysis, the size and quantity of spatters under different airflow velocities were examined.

When the airflow velocity is set to 0.6 m/s, as shown in Figure 11e, spatter behavior within the molten pool is significantly reduced. This is characterized by smaller spatter sizes and lower spatter counts. The reduced spatter activity is attributed to the effective action of airflow, which diminishes metal vapor generation and weakens the scattering effect of metal vapor and spatter on the laser beam, thereby enhancing spatter removal efficiency.

In contrast, when the airflow velocity is below 0.6 m/s, as illustrated in Figure 11a–d, spatter behavior intensifies as the airflow velocity decreases. The extent of metal vapor, droplet formation, and lateral spatter increases with diminishing airflow velocity. This is because lower airflow velocities result in reduced carrying capacity for metal vapor and spatter, leading to larger spatter sizes and higher spatter counts. Consequently, spatter re-deposition occurs on the molten pool and powder bed, causing localized powder layer thickness increases and the formation of large, poorly connected spherical particles [39].

Conversely, when the airflow velocity exceeds 0.6 m/s, as shown in Figure 11f, spatter behavior escalates. This is evidenced by enhanced metal vapor generation, more pronounced droplet and lateral spatter, and increased spatter scattering. At an airflow velocity of 0.75 m/s, spatter size and quantity significantly increase. This phenomenon is primarily due to the destabilization of the powder bed caused by excessively high airflow velocities, leading to powder movement and molten pool instability. Additionally, while high airflow velocities facilitate spatter removal, they also carry excessive metal vapor and spatter, potentially impinging on the molten pool and exacerbating spatter generation.

**Figure 12:**
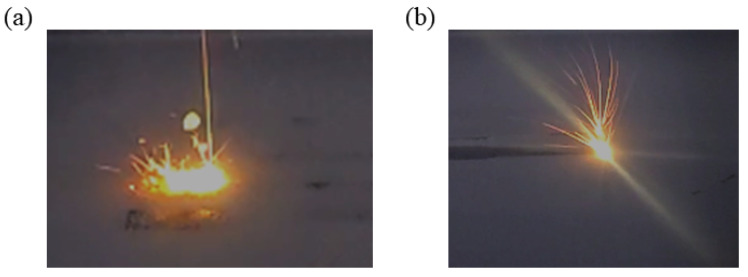
Images of the L-PBF process at different airflow velocities: (**a**) 0 m/s, (**b**) 0.6 m/s.

To further visualize the effect of airflow velocity on spatter removal, Figure 12 presents actual L-PBF process images captured at airflow velocities of 0 m/s and 0.6 m/s. The comparison reveals a significant improvement in spatter removal efficiency at the higher airflow velocity, suggesting that the formed parts under these conditions may exhibit superior mechanical performance. Consequently, the influence of airflow velocity on the mechanical properties of the formed parts is systematically investigated.

#### 3.2.2. The Effect of Airflow Velocity on the Performance of the Formed Part

Under different airflow velocities, the motion trajectories and removal efficiency of spatter particles exhibit significant differences, which directly influence defect formation and the final performance of the fabricated parts. To systematically elucidate the importance of effective spatter control for the quality of laser powder bed fused Inconel 718 components, this section discusses the influence of airflow velocity on key mechanical properties and microstructure through printing experiments conducted at varying flow rates. For this purpose, multiple indicators—including surface morphology, densification, microstructure, hardness, and tensile properties—were selected for comprehensive evaluation, so as to clarify the intrinsic relationship among gas flow velocity, spatter behavior, and part performance. The airflow velocity is shown in Table 6.

(1) Print quality analysis

**Figure 13:**
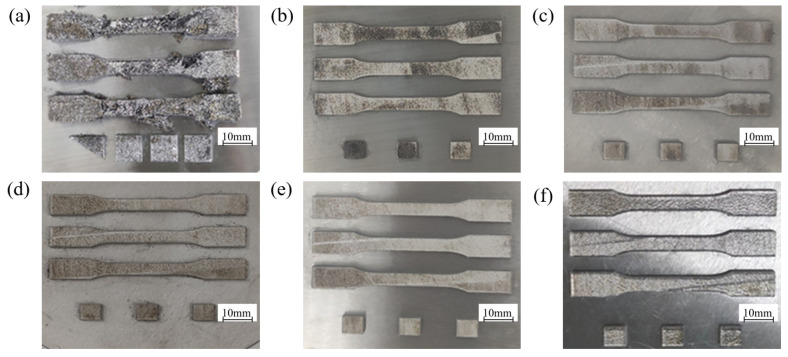
The surface of the formed part at different airflow velocities: (**a**) 0 m/s, (**b**) 0.15 m/s, (**c**) 0.3 m/s, (**d**) 0.45 m/s, (**e**) 0.6 m/s, (**f**) 0.75 m/s.

Three blocks and three groups of tensile parts were formed by L-PBF at each airflow velocity. The surface conditions of the formed blocks and tensile parts are shown in Figure 13.

**Figure 14:**
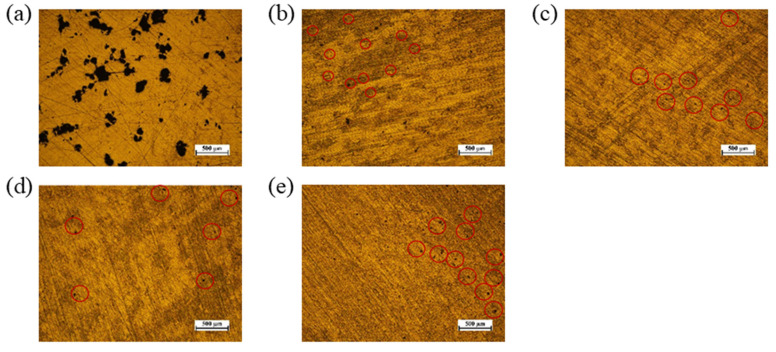
Observation of porosity on block surface at different airflow velocities: (**a**) 0.15 m/s, (**b**) 0.3 m/s, (**c**) 0.45 m/s, (**d**) 0.6 m/s, (**e**) 0.75 m/s.

To further analyze the impact of airflow velocity on the surface quality of the printed parts, metallographic microscope observations were conducted on the block surfaces. Due to the inability to complete printing without airflow, five parameters ranging from 0.15 m/s to 0.75 m/s were selected for subsequent examination, as illustrated in Figure 14. The redeposition of spatter particles is indeed a key mechanism leading to lack-of-fusion defects. When larger spatter particles fall back onto the powder bed, they introduce local irregularities and variations in thermophysical properties, thereby hindering uniform energy absorption and proper melt pool propagation during subsequent laser scanning, ultimately resulting in the formation of elongated, irregular pores [39]. As observed in Figure 14, the number of such elongated, irregular-shaped defects significantly increases when the airflow velocity deviates from the optimal threshold. This demonstrates that optimizing the airflow velocity can effectively reduce lack-of-fusion defects caused by spatter particle redeposition, thereby improving the internal quality of the fabricated parts.

**Figure 15:**
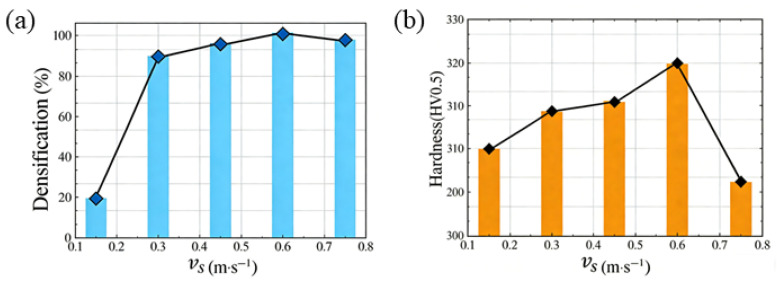
Print quality of laser powder bed fused Inconel 718 at different airflow velocities: (**a**) densification; (**b**) hardness.

Densification serves as a critical metric for evaluating the quality of L-PBF-formed samples under varying airflow velocities. Experimental densification data are presented in Figure 15a, illustrating the relationship between sample block densification and airflow velocity. The data reveal that at excessively low airflow velocities, sample density is significantly reduced. Conversely, under high airflow velocities, spatter is effectively removed, resulting in fewer internal defects and higher density. However, excessively high airflow velocities disrupt the powder bed, causing uneven powder layer thickness, internal defects, and a decrease in sample density. By analyzing the density and surface characteristics of samples across different airflow velocities, an initial optimal airflow velocity of 0.6 m/s is identified.

Hardness is another crucial metric for assessing the quality of L-PBF-formed samples under varying airflow velocities. This study conducted hardness tests at different airflow velocities, with results presented in Figure 15b. At low airflow velocities, insufficient powder melting leads to numerous internal pores with large sizes, resulting in low hardness. As airflow velocity increases, sample hardness improves due to enhanced density and uniformly refined microstructures. Higher airflow velocities stabilize the molten pool during L-PBF processing, enhance spatter dust removal efficiency, reduce internal defects, and refine the microstructure, further increasing hardness. However, excessively high airflow velocities disrupt powder bed stability, affecting powder layer thickness uniformity, causing spheroidization, increasing internal pores, and consequently reducing hardness.

(2) Mechanical performance analysis

**Figure 16:**
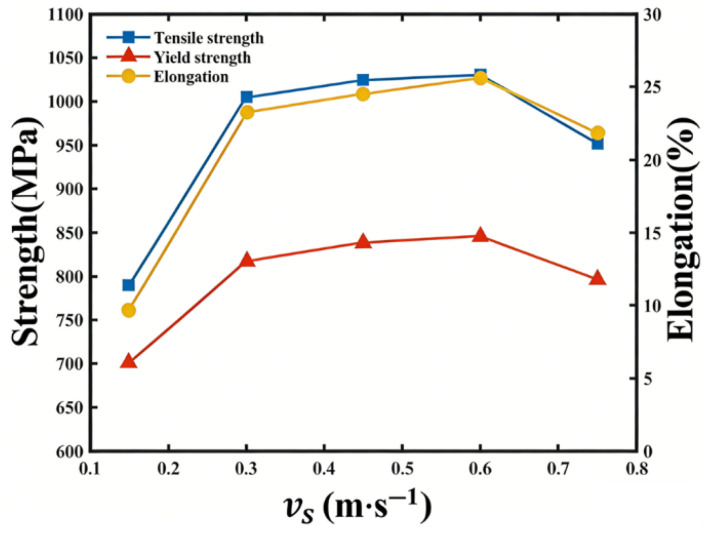
Mechanical performance of laser powder bed fused Inconel 718 at different velocities.

The Mechanical performance of the formed samples is evaluated through tensile strength, yield strength, and elongation tests, with results summarized in Figure 16. The experimental findings reveal that at low airflow velocities, the quality of L-PBF-formed tensile components is subpar, characterized by numerous internal pores and significantly reduced tensile strength, yield strength, and elongation. As airflow velocity increases, these mechanical properties begin to rise notably. However, excessively high airflow velocities disrupt powder bed stability, increasing internal pores and diminishing tensile strength, yield strength, and elongation. The optimal airflow velocity of 0.6 m/s yields the highest tensile strength, yield strength, and elongation among all samples. This experimental optimal airflow velocity is only 5% higher than the simulation-derived value, corroborating the findings from the previous simulation section.

## 4. Conclusions

To further enhance the performance of L-PBF-fabricated Inconel 718 parts, this study employs an integrated simulation–experimental approach to comprehensively analyze the influence of airflow velocity in the atmosphere protection system of L-PBF equipment—a parameter that has received relatively limited attention—on spatter motion behavior and removal effectiveness during the laser powder bed fusion process. Furthermore, the impact of airflow velocity on the performance characteristics of the fabricated parts is systematically investigated, with their properties correlated to spatter removal efficiency. Key findings and conclusions include the following:

(1) Computational Fluid Dynamics-Discrete Phase Model simulations identified an optimal airflow velocity of 0.57 m/s, considering both airflow uniformity and spatter removal efficiency at the upper substrate surface. Experimentally, high-speed camera observations of spatter behavior under varying airflow velocities yielded an optimal value of 0.6 m/s. The close agreement between the simulated and experimental values, with a marginal discrepancy of only 5%, validates the simulation approach.
(2) A self-developed high-speed microscopic imaging system was employed to investigate the motion behavior of spatter particles under different airflow velocities, revealing that the ineffective removal of spatter particles is a critical factor contributing to the formation of internal defects in the fabricated components. When the airflow velocity was below the optimum level, redeposition of spatter onto the molten pool and powder bed surface was observed, leading to the formation of pores and other defects in the as-fabricated parts. Conversely, when the airflow velocity was excessively high, both the size and quantity of spatter increased significantly, accompanied by a decrease in molten pool stability.
(3) The established correlation between effective spatter particle removal and the overall performance of additively manufactured components further demonstrates the critical role of optimizing the airflow velocity in the atmosphere protection system for part quality enhancement. At the optimal airflow velocity, the as-fabricated parts exhibit minimal internal porosity, resulting in superior surface quality, density, hardness, and tensile properties. Conversely, any deviation from this optimal velocity leads to increased pore formation, which subsequently deteriorates the quality of the fabricated parts.

Based on the present findings, future work will focus on extending this research in two key directions. First, the influence of gas flow velocity on the effective removal of machining-induced spatter in additive subtractive hybrid manufacturing processes will be investigated to optimize integrated process windows. Second, the validation and scaling of the observed effects on commercial multi-laser L-PBF platforms are planned to enhance the generalizability and industrial relevance of the established guidelines.

## Figures and Tables

**Table 1:** CFD simulation parameters.

Parameter	Symbol	Numerical Value	Unit
Argon density	$\rho_{k}$	1.63	kg/m^3^
Argon dynamic viscosity	$\mu_{k}$	2.13 × 10^−5^	kg/m·s
Average velocity of argon	U	1&2	m/s
Oxygen density	$\rho_{j}$	1.23	kg/m^3^
Oxygen dynamic viscosity	$\mu_{j}$	1.79 × 10^−5^	kg/m·s
Substrate temperature	T	150	℃
chamber pressure	P	0.43	mbar (gauge)

**Table 2:** DPM simulation parameters.

Parameter	Symbol	Numerical Value	Unit
Spatter density	$\rho_{p}$	1.55	g/cm^3^
Particle diameter	$d_{p}$	10~100	μm
Generation rate	$v_{p}$	10^−20^	kg/s
Build location	b	10	mm
Initial speed	$u_{p}$	1	m/s

**Table 3:** Chemical compositions of Inconel 718 powder.

E1	Ni	Cr	Fe	Nb	Mo	Ti	Al	Co	Si	Mn	Cu	C	P	N	B
wt%	53.70	17.93	18.17	5.20	2.96	0.95	0.48	0.33	0.08	0.08	0.05	0.025	0.009	0.004	0.0025

**Table 4:** The average airflow velocity of the upper surface of the substrate at different parameter settings.

Scheme	$v_{a}$/m·s^−1^	$v_{i}$/m·s^−1^	$v_{s}$/m·s^−1^
a	1.0	0.5	0.26
b	1.0	1.0	0.46
c	1.0	2.0	0.78
d	2.0	1.0	0.57
e	2.0	1.5	0.74
f	2.0	2.0	0.92

**Table 5:** Standard deviation of airflow velocity for different schemes.

$v_{s}/$m·s^−1^	0.26	0.46	0.78	0.57	0.74	0.92
Standard deviation	0.060	0.078	0.081	0.047	0.040	0.065

**Table 6:** Experimental airflow velocity design of different schemes.

Scheme	a	b	c	d	e	f
$v_{s}/$m·s^−1^	0	0.15	0.30	0.45	0.60	0.75

## Data Availability

The original contributions presented in this study are included in the article. Further inquiries can be directed to the corresponding author.
